# Malocclusion and rhinitis in children: an easy-going relationship or a yet to be resolved paradox? A systematic literature revision

**DOI:** 10.1186/s13052-018-0537-2

**Published:** 2018-08-22

**Authors:** Francesca Occasi, Ludovica Perri, Matteo Saccucci, Gabriele Di Carlo, Gaetano Ierardo, Valeria Luzzi, Giovanna De Castro, Giulia Brindisi, Lorenzo Loffredo, Marzia Duse, Antonella Polimeni, Anna Maria Zicari

**Affiliations:** 1 Department of Pediatrics, “Sapienza” University of Rome, Rome, Italy; 2 Department of Pediatric Otorhinolaryngology, “Sapienza” University of Rome, Rome, Italy; 3 Department of Internal Medicine and Medical Specialities, “Sapienza” University of Rome, Rome, Italy

## Abstract

**Objective:**

The relation between nasal flow and malocclusion represents a practical concern to pediatricians, otorhinolaryngologists, orthodontists, allergists and speech therapists. If naso-respiratory function may influence craniofacial growth is still debated. Chronic mouth-breathing is reported to be associated also with a characteristic pattern of dental occlusion. On the other hand, also malocclusion may reduce nasal air flows promoting nasal obstruction. Hereby, the aim of this review was to describe the relationship between rhinitis and malocclusion in children.

**Methods:**

An electronic search was conducted using online database including Pubmed, Web of Science, Google Scholar and Embase. All studies published through to January 30, 2017 investigating the prevalence of malocclusion in children and adolescents (aged 0-20 years) affected by rhinitis and the prevalence of rhinitis in children with malocclusion were included. The protocol was registered at PROSPERO - International prospective register of systematic reviews under CRD42016053619.

**Results:**

Ten studies with 2733 patients were included in the analysis. The prevalence of malocclusion in children with rhinitis was specified in four of the studies ranging from as high as 78.2% to as low as 3%. Two out of the studies reported the prevalence of rhinitis in children with malocclusion with a rate ranging from 59.2 to 76.4%.

**Conclusion:**

The results of this review underline the importance of the diagnosis and treatment of the nasal obstruction at an early age to prevent an altered facial growth, but the data currently available on this topic do not allow to establish a possible causal relationship between rhinitis and malocclusion.

## Background

The relation between nasal flow and malocclusion has been pondered over for decades. No consensus has yet been reached concerning how nasal-respiratory function may influence craniofacial growth [1]. The interest of orthodontists in the seventies focused on the airways and lead to recommendation of removal tonsils and adenoids in order to improve facial growth and mode of breathing. The significant change in growth after adenoidectomy demonstrated by Linder-Aronson [2, 3] have had a high impact on the orthodontic community [4–9]. The link between mode of breathing and development of malocclusion resulted in the “soft-tissue stretching hypothesis” by Solow and Kreiborg [10]. Indeed, a change in jaw posture or a change in head posture could lead to stretching of the lips, cheeks and musculature of the face resulting in upright incisors and narrower dental arches, which often are observed in patients with long face and open bite growth pattern.

Chronic oral breathing in children, usually due to adenoid hypertrophy or chronic rhinitis, is associated with maxillary deficiency and excessive development of the vertical axis of the facial skeleton, the exact measure of the effect of this complicated relationship is still unknown. This issue represent a great practical concern to pediatricians, otorhinolaryngologists, orthodontists, allergists and speech therapists. Interestingly a cross-sectional study from Grippaudo et al.carried out on 3017 children showed that bad habits and mouth breathing were associated with more severe malocclusions [11]. Moreover, chronic mouth-breathing is reported to be associated with a characteristic pattern of dental occlusion. On the other hand also malocclusion may contribute to reduce nasal air flows mechanically promoting nasal obstruction. Hence, the direction of this pathogenic process might be difficult to identify and flow charts often used to schematize the role played by nasal obstruction on the growth of the craniofacial district are too simplified to ascribe to the process its complexity.

**Fig. 1:**
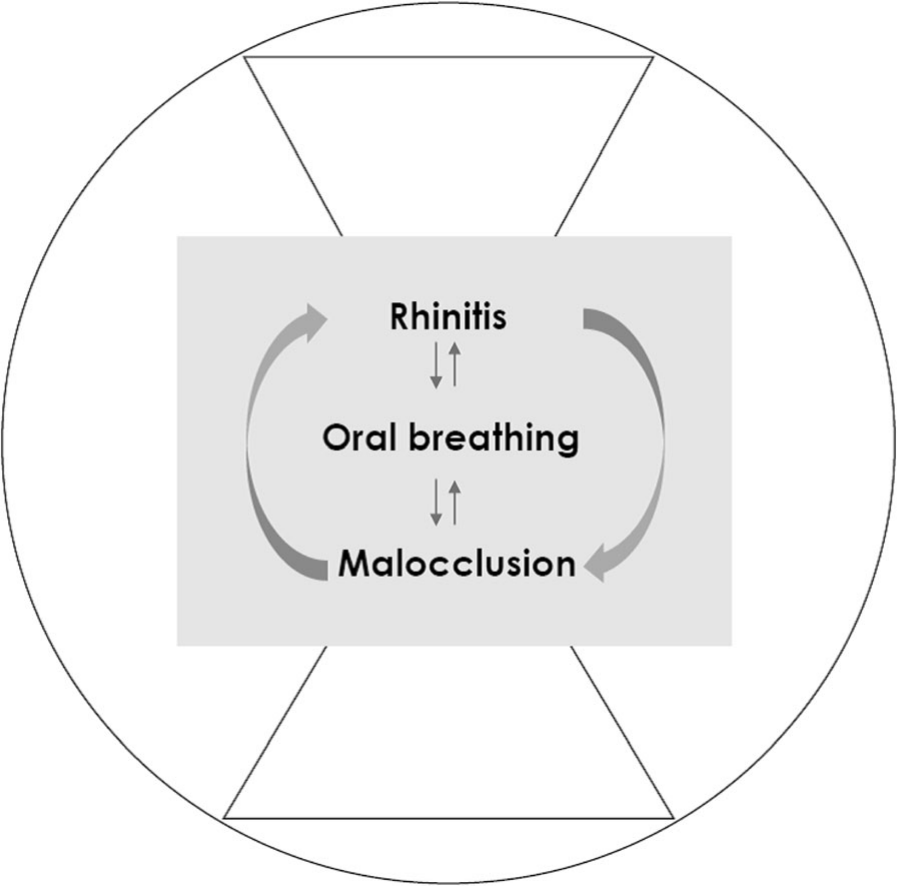
Malocclusion and Rhintis are to some extent concomitant disorders

Hereby, the aim of this review is to describe the relationship between rhinitis and malocclusion in children (Fig. 1). A systematic literature revision including studies on children and adolencents assessing the prevalence of rhinitis in children with malocclusion and the prevalence of malocclusion in children with rhinitis was performed.

## Material and methods

The protocol was registered at PROSPERO - International prospective register of systematic reviews (Centre for reviews and dissemination, University of York, York, United Kingdom) under CRD42016053619.

Articles reporting class of malocclusion according to Angle's classification system [12] or general prevalence of malocclusion, unless otherwise specified, in the main text or in the tables were considered in this analysis.

Patients that showed nasal signs and symptoms of chronic inflammation of the nasal lining mucosa such as nasal congestion, rhinorrhea, sneezing and itching were considered affected by rhinitis. Diagnosis of allergic rhinitis (AR) was based on clinical history, data relative to allergic rhinitis collected through questionnaires, clinical examination by the allergist and a positive response to the allergy test.

### Inclusion and exclusion criteria

This systematic review only included articles that investigated the prevalence of malocclusion in children and adolescents (aged 0-20 years) affected by rhinitis and the prevalence of rhinitis in children with malocclusion. Considering rhinitis and, in particular AR, as one potential cause of mouth breathing and primary snoring also original researches including a sample selected or mouth breathing and primary snoring but reporting rhinitis as the main cause were included. Any study type between 2000 and 2017 with relevant cross-sectional data was included.

Articles in languages other than English, Italian, French, Spanish were excluded.

### Information sources and search

An electronic search was conducted using online database including Pubmed, Web of Science, Google Scholar and Embase. All studies published from January 1, 2000 through to January 30, 2017 were included. The last search was run on March 9, 2017. The reference lists of the selected articles were manually screened to identify any additional references not found during the search of the electronic databases. Articles obtained from consultations with experts in the field were also considered. The detailed search strategies were prepared following the consultation of an expert bibliographer. The following string has been used: (("rhinitis" [MeSH Terms] OR "rhinitis" [All Fields]) OR ("nasal obstruction" [MeSH Terms] OR ("nasal" [All Fields] AND "obstruction" [All Fields]) OR "nasal obstruction" [All Fields])) AND ("malocclusion" [MeSH Terms] OR "malocclusion" [All Fields]).

### Study selection

A two-phase process to select the final articles was followed. In phase 1, titles and abstracts were independently screened of all the gathered references. All articles that did not meet the inclusion criteria were excluded. In Phase 2 articles were considered in their full text. The inclusion criteria were as follows: children and adolescents, studies with cephalometry or clinical orthodonic evaluation. Furthermore, the following exclusion criteria were used: animal studies, in vitro studies, case reports, reviews, and studies including participants with craniofacial syndromes or receiving orthodontic treatments before evaluating.

### Data collection process and quality assessment

The following data were extracted from the studies: total number of patients included, number of patients with AR included, number of patients with malocclusion included, age, gender distribution, prevalence of malocclusion in patients with or without rhinitis, prevalence of rhinitis in patients with or without malocclusion, primary end-point of the study.

## Results

**Fig. 2:**
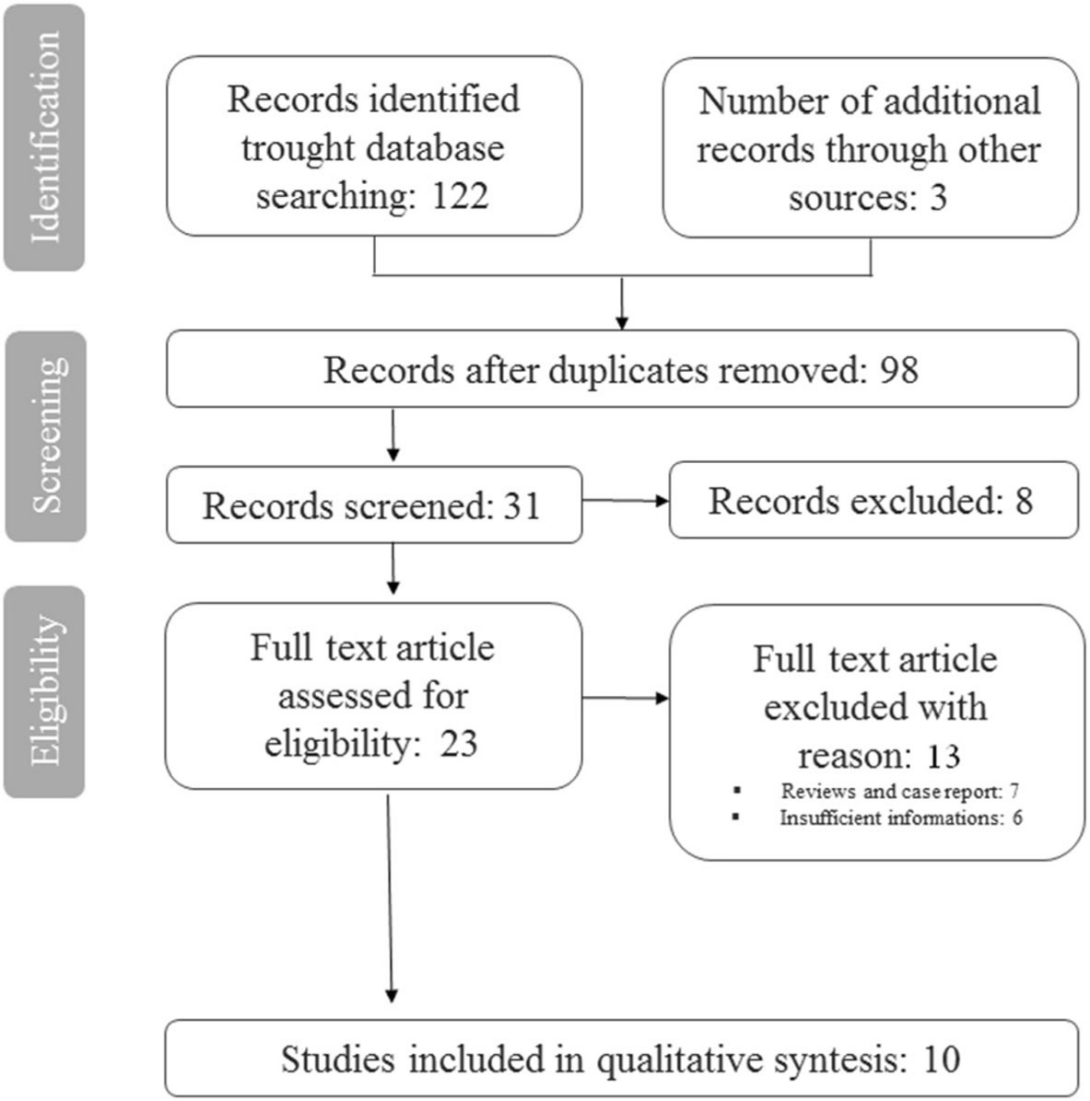
Study selection

The search provided a total of 125 citations. Of these, 98 remained after exclusion of duplicates. Of these, 67 studies were discarded after reviewing the titles and abstracts because it appeared that these papers clearly did not meet the selection criteria. We obtained 31 records; 8 of these were not full text article and were excluded. 23 full text article were assessed for eligibility. Of these 13 were discarded for other reason, 7 were review articles and case reports and 6 were discarded for insufficient informations. A total of 10 studies [13–22] met the inclusion criteria and were included in this systematic review. The study identification and selection progression is summarized in Fig. 2.

All studies were written in English. The 10 studies ranged in size from 30 to 1160 patients for a total of 2733 patients (Tables 1, 2). Clinical characteristics of the study populations are illustrated in the Tables 1 and 2. Compared to females, males were more frequently distributed in all trials (Tables 1, 2), only four trials did not reported male/female distibution. The rate of AR (AR+) was 29.5% among the studies. The prevalence of malocclusion in children with AR was specified in three of the studies [13, 16, 17] ranging from as high as 78.2% to as low as 43.1% (Table 1). Only one of the studies reported the prevalence of MO in patients with non-AR that was 7.36% [18]. The rate of children with malocclusion (MO+) was 38.2% among the studies. Two out of the studies [21, 22] reported the prevalence of AR in children with malocclusion with a rate ranging from 59.2% to 62.9% (Table 2). The rate of non-AR in patients with malocclusion was reported only in one of the studies and was 76.4% [22].

**Table 1:** Clinical characteristics of the studies evaluating malocclusion in children with rhinitis

Study	Total number of patients	Number of patients with AR *n* (%)	Number of patients with non-AR *n* (%)	Age (yr)	M/F	MO in AR+ *n* (%)	MO in non- AR *n* (%)	MO in AR- *n* (%)	Primary end-point
Vazquez 2006	1160	334 (28.8)	-	4-5	582/578	144 (43.1)	-	496 (60)	Malocclusion in the primary dentition
Souki 2009	401	75 (18.7)	326 (81.3)	2-12	-	-	24 (7.36)	13 (4)	Class II malocclusion
Sundus M. Bezzo 2005	300	13 (4.3)	-	5-19	176/124	29 class II (AR &/or asthma) 10 class III (AR &/or asthma)	-	22 class II (AR &/or asthma) 7 class III (AR &/or asthma)	Effect of oral respiration on the developing dentition in patients with respiratory tract allergies
Agostinho 2015	70	35 (50)	-	5-14	41/29	-	-	-	Dental positions, skeletal effects and the pharyngeal airway space
De Freitas 2001	192	101 (52.6)	-	2-12	-	-	-	-	Transverse and vertical palate dimensions
Ghasempour 2009	100	50 (50)	-	3-12	-	-	-	-	Palatal arch diameters
Harari 2010	116	55 (47.4)	-	10-14	54/62	40 (72.7) class II 3 (5.4) class III	-	38 (62.3) class II 4 (6.55) class III	The effectof mouth breathing during childhood on craniofacialand dentofacial development
Zicari 2014	30 (PS)	-	-	4-8	14/16	-	-	-	Correlation between rhinomanometric and cephalometric parameters in children with primary snoring (PS)

*AR* Allergic rhinitis, *MO* malocclusion

**Table 2:** Clinical characteristics of the studies evaluating rhinitis in children with malocclusion

Study	Total number of patients	Number of patients with MO *n* (%)	Age (yr)	M/F	AR+ in MO *n* (%)	non-AR in MO	AR- in MO *n* (%)	Primary end-point
Luzzi 2013	275	125 (45.4)	5-9	126/149	74 (59.2)	-	20 (16)	Association between allergic rhinitis and malocclusions in primary and early-mixed dentition
Imbaud 2015	89	89 (100)	8-15	-	56 (62.9)	68 (76.4)	21 (23.6)	Frequency and etiology of rhinitis, oral breathing, types of malocclusion and orofacial disorders in patients treated for dental malocclusion

*AR* Allergic rhinitis, *MO* malocclusion

### Malocclusion in children with rhinitis

Many authors investigated the type of occlusion and the prevalence of malocclusion in childrenwith nasal obstruction using cephalometric analysis. Study characteristics are summarized in Table 1. Concerning general prevalence of malocclusion in children with AR (AR) Vazquez et al. [13] did not find a significant difference between controls and the case group (55.1% vs 55.4%) although AR and AR + NNSH (Non Nutritive Sucking Habits) were associated with malocclusion as determined by logistic regression model including all children.

In 2001 de Freitas [14] found no statistically significant differences between children with persistent AR and the control group in terms of intercanine and intermolar distance. In addition, Ghasempour [15] did not find a significant difference between the case and the control group for intermolar and intercanine distances in both primary and mixed dentition. On the other hand, significant narrowing of both upper and lower arches at the level of canines and first molars were found in the mouth breathers group [16].

In 2005 Bezzo et al. [17] found an high prevalence of class II and III malocclusion in children with AR while concerning posterior and anterior cross-bite, the difference did not reach statistical significance, although an higher percentage of children was found in the group with persistent AR (9.5% vs4.4% and 6.8% vs 2.7%). Harari et al. [16] found in children with nasal obstruction that class II was three times more common than class I and that posterior crossbite was significantly more frequent in cases (49%) than controls (26%), although he could not find any significant difference in malocclusion classification between groups.

The importance of posterior cross-bite was highlighted also by Vazquez Nava et al. [13] However, study results showed that the effect of NNSH and bottle feeding on posterior cross-bite might be more important than AR in determining malocclusion. In line with this results, Ghasempour [15] found an higher prevalence of cross-bite in children with AR when compared to healthy controls. Concerning palate dimensions, in the primary dentition phase depth resulted 1.5mm greater in allergic children than in controls and with an average of 1.7 mm in the mixed dentition phase [14]. Of note, many discrepancies were found by different authors concerning overjet and overbite. Bezzo et al. [16] could not find any significant difference while Vasquez [13] reported that AR alone and together with NNSH was associated with anterior open bite by a logistic regression model although he found open bite in 52.3% children with rhinitis and 50.7% children without rhinitis. On the other hand, Ghasempour [15] found a deeper palate depth in both primary and mixed dentition when comparing cases and controls. Bezzo et al [17] also reported a higher prevalence of tooth crowding in allergic patients compared to healthy controls.

Cephalometric analysis showed a significant increase in the mandible plane angle, an increased y-axis angle and a higher palatal plane [16]. Moreover, a larger A point-Nasion-B point (ANB), Frankfurt-mandibular plane angle, sella nasion line and occlusal plane angle with the allergic individuals being more vertical [19] was reported. Of note, allergic children presented smaller maxillas, measured between Condilium and A point (Co-A) as maxillar length and Gnation (Co-Gn) as mandibular length. Also the Ricketts anterior-inferior skeletal height was statistically different between groups and the most significant difference was an increase in inferior facial height [19].

Moreover, concerning pharynx dimensions in allergic children compared to normal nasal breathing children a statistically significant decrease in many distances were found including the distance between posterior nasal spine and posterior wall of pharynx, superior McNamara airway space, the space between soft palate and posterior wall of pharynx, the distance between inferior soft palate point and posterior wall of pharynx and inferior McNamara airway space [19].

Interestingly, significant correlations were found between nasal flows and ANB, posterior rotation of the jaw and vertical growth, inferior divergence, horizontal growth, posterosuperior airway space [20]. Moreover, also a reduction of postero-superior pharyngeal lumen in patients with a lower nasal patency was highlited [20].

Concerning anterior oral seal (AOS), lip to lip AOS appeared to be normal in most of nose breathers (70%) while abnormal lip-to-tongue AOS was significantly more frequent in the mouth breathers group (56%) than nose breathers group [16].

In the work by Souki et al. [18] only 42% of the children with non-AR selected for mouth breathing presented with a sagittal disharmony, represented by class II or III and that the prevalence of class III became higher as kids get older. Also Souki et al. [18] reported an higher percentage of open bite in children with mouth breathing when compared to general population (29.2% vs 12-20%) in children with mixed dentitions and a higher prevalence of cross-bite was found in mouth breathing children than in the general population (close to 30% during deciduous and 48% in permanent dentition vs 22% and 3.9%).

### Rhinitis in children with malocclusion

On the other side, the relationship between dental occlusion and rhinitis could be investigated also assessing the prevalence of rhinitis in children with malocclusion. However, few authors investigated this issue from this point of view. Study characteristics are summarized in Table 2.

In 2013 Luzzi V. et al. [21] carried out a case control study including 125 individuals affected by malocclusions and 150 healthy patients, finding that children with a history of AR had a threefold increased risk to develop one or more dento-skeletal alterations. Moreover, significant associations were found between AR and the development of posterior crossbite and increased overjet. They concluded that AR is a significant risk factor for the development of malocclusions and is associated with the development of posterior crossbite and increased overjet.

Also in children with non-AR Imbaud et al. [22] found in patients undergoing orthodontic treatment a prevalence of rhinitis of 76.4% whereas the frequency of oral breathing was 62.9%. The authors underlined that the frequency of rhinitis in children with dental malocclusion is higher than that in the general population, which is approximately 30%. Moreover, patients with oral breathing have a tendency to a dolichofacial growth pattern (increased Y axis of facial growth). In patients with rhinitis, regardless of the presence of oral breathing, the dolichofacial growth tendency was not observed.

## Discussion

The effects of oral breathing on nasomaxillary growth in childhood were already debated by Hippocrates back in the 5th century B.C. [23].

Moreover nasal breathing allows proper growth and development of the craniofacial complex interacting with other functions such as mastication and swallowing [24]. Hence, a normal craniofacial growth seems to depend on physiological nasal breathing as confirmed also by experimental models [23] where a progressive decrease in height, width and length of the skull of a rat was recorded proportionally to the entity of nasal obstruction.

Craniofacial growth changes at various periods were described by several authors [25–34]. Growth of various parts of the head neither proceeds at the same rate nor follows the same pattern [35, 36]. A previous study reported that indices of upper face width remain on average constant between 5 to 11 years and the corresponding indices for the lower face increase from 80% at 5 years to 82% at age 11 [37]. Snodell et al. evaluated longitudinal normal growth changes in the transverse and longitudinal dimension between 4 and 20 years and found that vertical growth is greater than transverse growth [38]. Bishara et al. described the changes that occured in the face between 5 and 25 years and between 25 and 45 years and found a significant increase in overbite in females but not in males between 25 and 45 years [39, 40]. Changes at later stages of maturation were assessed only in few studies because the facial growth is thought to be complete by late adolescence. For example Bishara et al. relate changes in overbite over a 40-year span with those occurring in vertical skeletal facial relationship and found no significant association although overbite changes were significantly associated with changes in some vertical skeletal parameters [41].

Other studies described the large variations of the human face [41]. Ligthelm-Bakker et al. [42] found a negative correlation between the average growth rate of the upper anterior face height with the lower anterior face height. Tsunori et al. [43] reported that facial type (short, average and long faces) in relation to morphological characteristics is an important factor to be considered in orthodontic treatment, because facial type influences growth prediction of the maxillofacial system in the anchorage system used during orthodontic treatment. Facial proportions and height, once defined, stay constant throughout the life.

The success of an orthodontic treatment is not only dependent on understanding where craniofacial growth occurs, but also when it ends [44]. The vertical component of growth is thought to be the last to end, so failure to control it may compromise the results and cause relapse after treatment [45, 46]. So an accurate assessment of such discrepancies in the vertical facial pattern could ensure the treatment success [47].

However, there is another side of the issue that needs to be debated. Of note, also maxillary constriction and a high palatal vault with the elevation of nasal floor may lead to a mechanical decrease in nasal airway size [26]. The change in the muscular function and the occurrence of orthodontic anomalies may cause alteration in patients’ breathing habits [48].

Moreover, most of chronic mouth-breathing can be caused by chronic nasal obstruction, adenoidal hypertrophy (AH), or anatomic abnormalities such as cleft palate and it may be a learned habit as well [49].

Nasal obstruction may be often underestimated and it is a frequently encountered problem in the pediatric age and the child cannot obviously recognize the cause of the airflow impairment and nasal obstruction [50]. Moreover, although, in the toddler, nasal obstruction is frequently considered indirectedly caused by AH, it should be pointed out that it could depend on other relevant causes such as respiratory infections and allergy representing the two most relevant inflammatory conditions associated with nasal obstruction in children. AR is frequent in children, affecting up to 30% of the general population, and may also cause the open-mouth posture and the so-called “adenoidal facies” commonly attributed to AH [51].

AH is detected in 1/3 of the general pediatric [52] and large adenoids may be associated with absence of allergy. Ameli et al. [51] found relevant adenoid hypertrophy, such as grade 3 and 4, in 1/3 of allergic children while, on the other hand large turbinates may be associated with small adenoids, hence, underlining again the importance of a detailed evaluation of the nose and the rhinopharynx.

Concerning AR also the role of perennial rather than seasonal allergens in inducing malocclusion needs to be considered. In the study from Luzzi et al. [21] data about type of AR and allergens to which the child was most sensitive (seasonal AR, i.e., caused by exposition to grass or pollens, or perennial AR, i.e., caused by ex position to dust mites or animal dander) were reported. In this study among allergic children with malocclusion, 60% were sensitive to a single allergen and 40% to more than one allergen. Among allergic children without malocclusions, 90% were sensitive to a single allergen and only 10% to more than one allergen. Allergic children in the case group show a high prevalence of perennial allergy (73%), both alone (41%) or in association with seasonal allergy (32%), whereas children in the control group are mostly affected by seasonal allergy alone (70%). This study also confirms that children affected by AR had a threefold increased risk to develop dento-skeletal alterations. In the study of the De Freitas et al. only children with perennial allergic rinitis (AR) were recruited [14]. In this study no significant difference of the transverse dimension of the palate were found between case and control groups. Another recently published paper also demonstrated that malocclusion seems not to be a co-morbidity of AR due to pollens [53].

The aim of the present review was to assess the relationship between rhinitis and malocclusion in children. Considering that this issue has been the object of several debates over decades since the 20th century, the choice of including only manuscripts since 2000 came from the need of resuming the last evidence based on current validate diagnostic methods. Moreover, the literature of the last years clearly reflects finding of the previous century and takes over from the first researches on the topic.

The article selection represented one of the main problems of this revision. Actually, most of original researches found were focused on mouth breathing, considered as a potential consequence of rhinitis while only in few studies children were selected for rhinitis tout court. For this reason we chose to include also artiche selecting children for other reasons than rhinitis such as mouth breathing and primary snoring but clearly reporting that the diagnosis were related to rhinitis and nasal obstruction for most of the sample. For example, Souki et al. [18] reported that the overall prevalence of AR was 72.1% and hence the study was included. Similarly, the studies by Zicari et al. [20] and Imbaud et al. [22] were included in the revision.

Another element of the revision to be pointed out is that results were divided in two sections: malocclusion in children with rhinitis and rhinitis in children with malocclusion. This decision was related with the need of investigating the issue from two different point of views. It should be underlined that most of the studies were conducted by dental researchers and hence, patients were selected for malocclusion or at for a suspect of malocclusion. Moreover, most of study groups conducted a strict analysis on dental and facial characteristics but investigated the eventual presence of rhinitis through questionnaires and reported medical advices without reporting ARIA (Allergic Rhinitis and its Impact on Asthma) classification or performing objective test such as rhinomanometry or nasal cytology. In this perspective, it is important to underline that children often underestimate their nasal symptoms [50] and, hence, especially from a pediatric perspective, most of the included studies report a selection and allocation bias. This might be regarded as the main limitation of the present review. In fact, the prevalence of almost 77% of children aged 8-15 years with malocclusion affected by rhinitis, although higher when compared to the prevalence of 30% of children affected by rhinitis in the general population, might be over or, most probably, still down estimated.

Another potential limitation of this review is that the research was conducted since 2000. However, results were in line with previous evidence and offered further information and details. In this perspective, it should be underlined that already in eighties many authors reported that children with AR [52] have longer faces with narrower maxillae, lower facial height, smaller angle of the mandibular incisor to the mandibular plane, larger palatal height and retrognathic jaws.

Another interesting issue to assess the relationship between AR and malocclusion might be the evidence of the potential effect of rhinitis treatment on malocclusion and of the potential effect of dental occlusal treatment and maxillary expansion on nasal airflow.

Consequences of orthodontic treatment on nasal breathing was assessed by several authors. Already Hershey et al. [54] reported that Rapid Maxillary Expansion (RME) provides a 45% reduction of nasal resistance as well as significant widening of nasal passages. The RME treatment is able to induce more pronounced transverse craniofacial changes at the skeletal level when the subjects were treated before the peak in skeletal maturation. Besides expanding the maxilla, RME is effective in increasing the minimum cross-sectional area of nasal cavity, which is highly responsible for nasal resistance. In 2006, Enoki C et al. [55] conducted a prospective longitudinal study on 29 patients, ranging from 7 to 10 years of age with oral breathing. Interestingly, no significant difference in minimal cross-sectional area, neither in the region of the nasal valve nor in the inferior turbinate at the three time points studied was found, although mean nasal resistance were significantly lower after treatment than before. These results are in line with previous results [56] detecting a reduction in nasal resistance after expansion.

In a previous study Grippaudo et al.conducted a survey to estimate the prevalence of malocclusions and to assess whether the severity of malocclusions could be modified during the mixed dentition phase towards the full permanent dentition. In this study Class III malocclusion, moderate or severe crossbites and severe increased overjet and overbite seem not to improve spontaneously so early treatment of these orthodontic discrepancies is intended to avoid the development of more severe discrepancies in the late mixed and permanent dentitions and can shorten the treatment time or eliminate the need for treatment at a later age [57].

Concerning the potential effect of treatment of rhinitis on malocclusion, most of authors investigated the effect of adenoidectomy and tonsillectomy on dental occlusion. In 2011 Pereira SR et al. [58] found that adeno-tonsillectomy was effective in improving some dental measurements, with benefits to growing patients preventing malocclusions from becoming difficult to treat or permanent. In addiction, Zhu Y. et al. [59] aimed at determining the effect of adenoidectomy and tonsillectomy on the growth of dental morphology in children with airway obstruction with a systematic review focused on 8 articles. In terms of dental arch with, malocclusion, palatal height, overjet, overbite, dental arch perimeter, and arch length, a tendency toward normalization was evident following adenoidectomy or tonsillectomy concluding that following adenoidectomy and tonsillectomy, the malocclusion and narrow arch width of children with airway obstruction could not be completely reversed.

## Conclusion

This review confirms that no true relation exists between maloccusion and rhintis and vice-versa. Probably are each other only comorbidities or concomitant disorders. In fact rates of association between the two conditions are of 29.5 and 38.2% respectively.

The importance of the nasal obstruction diagnosis and treatment at an early age to prevent an altered facial growth and abnormal perioral muscle function is fascinating although many questions are still waiting for answers. The critical age for the onset of nasal obstruction, the time of the persistence of rhinitis before a growth effect appear needs to be clarified. Moreover, studies investigating the effect of rhinitis treatment on dental occlusion and facial morphology in preschool children should be strongly encouraged.
